# Effects of impaction severity of treated palatally displaced canines on periodontal outcomes: a retrospective study

**DOI:** 10.1186/s40510-018-0256-7

**Published:** 2019-02-04

**Authors:** Alberto Caprioglio, Ilaria Comaglio, Lea Siani, Rosamaria Fastuca

**Affiliations:** 1 Department of Medicine and Surgery, University of Insubria, Varese, Italy; 2 Private Practice, Pavia, Italy; 3 Department of Medical, Surgical and Health Sciences, University of Messina, Messina, Italy; 4 C/O Dental School, Via G. Piatti, 10, 21100 Messina, Velate (VA) Italy

**Keywords:** Orthodontic treatment, Tooth, Impacted

## Abstract

**Background:**

Even though periodontal health was suggested to be not related to the traction technique, some other variables might influence the esthetic outcome of palatally displaced canines (PDC) when aligned, such as the initial position and impaction rate of the canine before treatment. The purpose of the present study was therefore to evaluate the existing correlations between periodontal health outcome of PDC after their exposure and alignment and their initial position identified according to the different rates of impaction severity.

**Materials and methods:**

The final sample enrolled 293 PDC which satisfied inclusion and exclusion criteria. All the canines were exposed using CT by the same oral surgeon and orthodontic traction was applied using the easy cuspid device followed by fixed appliance treatment. Image analysis and periodontal status evaluation were performed for all PDCs.

**Results:**

*α*-Angle and *d*-distance showed no significant differences in the periodontal outcome of PDCs after treatment. The only tested variable showing significant differences was *S*, since canines with CEJ visible at the end of the treatment presented sectors with a mean score of 1.67, which was significantly different (*P* < 0.05) when compared to the *S*-sector for the canines that showed PD < 2 mm at the end of the treatment.

**Conclusions:**

Radiographic variables as *α*-Angle and *d*-distance seem to not influence the periodontal outcome of the treated impacted canine regardless of the amount of gravity. On the contrary, *S*-sector might play a significant role when higher rates of gravity are present suggesting the possibility in few cases for periodontal damage at the end of treatment.

## Background

Maxillary canine is the tooth most frequently involved in eruption problems after the third molar, and canine displacement was considered as an eruptive disorder and was suggested as a precursor of canine impaction [1]. The prevalence of impacted canines in the orthodontic population was reported between 0.9 and 2.2% [2], and in most of cases, the canines are ectopically positioned. Multiple etiological factors were linked to canine impaction as absence of lateral incisors, anomalies of lateral incisors, lack of guidance, ectopic position of the tooth germ, presence of obstacles to eruption, or genetic factors [3]. The displacement was suggested with higher prevalence on the palatal side [4] and often associated to lack of space in the dental arch when labial displacement was observed [5] then palatally displaced canine (PDC) was the condition more frequently observed.

Treatment of PDC might be performed with early interceptive measures [6–9], when applicable, or late treatment that might lead to combined surgical-orthodontic treatment to relocate the canine into the arch with orthodontic alignment [10, 11]. Autotransplantation [12] and extraction [13] were also suggested as alternative approaches.

When surgical exposure of the canine’s crown and its orthodontic alignment is performed, the treatment might lead to different periodontal health status and often be related to damage of the periodontal supporting structure due to the forced orthodontic eruption of the tooth [14]. As a matter of fact, ectopic impacted canines showed increased plaque and gingival bleeding index, greater pocket depths, reduced attached gingival width, higher gingival levels, increased crown lengths, higher electric pulp testing scores, and reduced bone levels at the end of orthodontic treatment compared to their contralateral teeth with physiologic eruption [15, 16].

Mainly two methods of surgical exposure of the canine were previously described [10, 11] comprising open eruption technique (OT) and closed eruption technique (CT). The former technique usually requires the removal of the soft tissue and bone covering the crown of the canine followed by the optional application of a surgical pack, then leaving the impacted canine to spontaneously erupt or bonding an orthodontic attachment on the crown to apply traction [17]. In this case, healing would take place by second intention [18]. Among the advantages of the OT direct vision for the orthodontist and visual control of the canine movement during treatment [19], less time for surgery, few cases of reprocessing, better hygiene during treatment, and healthy periodontal tissues after treatment were previously described [10, 11].

The CT, on the contrary, always involves raising a full mucoperiosteal flap to expose the canine crown with the bonding of an orthodontic attachment. Then the flap is usually repositioned and the orthodontic traction is applied after complete healing [10, 11]. In some cases, the simultaneous removal of the deciduous canine might be performed with the aim of creating a tunnel where the impacted canine can be easily conducted [20]. Reduction of intraoperative bleeding and better patient comfort during the healing process were suggested as benefits of the CT [19].

According to the results of recent systematic review and meta-analysis [11], OT was found to be faster in treatment duration and subjected to lower risk of ankylosis compared to CT. Nevertheless, no differences between the two techniques were found regarding canine esthetics and periodontal health outcomes [10]. Even though periodontal health was suggested to be not related to the traction technique, some other variables might influence the esthetic outcome of the canine when aligned, such as the initial position and impaction rate of the canine before treatment.

The purpose of the present study was therefore to evaluate and compare the existing correlations between periodontal health outcome of PDC and their initial position identified according to the different rates of impaction severity in adolescent patients after their exposure and alignment. The null hypothesis was that no direct correlation exists between the periodontal health of a treated PDC and its initial rate of impaction, since some other factors might play a key role as surgical technique or treatment management.

## Material and methods

The study sample was selected by retrospective screening of 438 impacted PDC of 406 patients treated by the same expert operator (AC) between January 2001 and January 2018, archived in private practice. As a routine procedure, a signed informed consent for releasing diagnostic records for scientific purposes was obtained from the parents of the patients prior to entry into the treatment. The protocol was reviewed and approved by the Ethical Committee of the University of Insubria (Varese, Italy) (approval no. 725) and procedures followed adhered to the World Medical Organization Declaration of Helsinki.

**Fig. 1:**
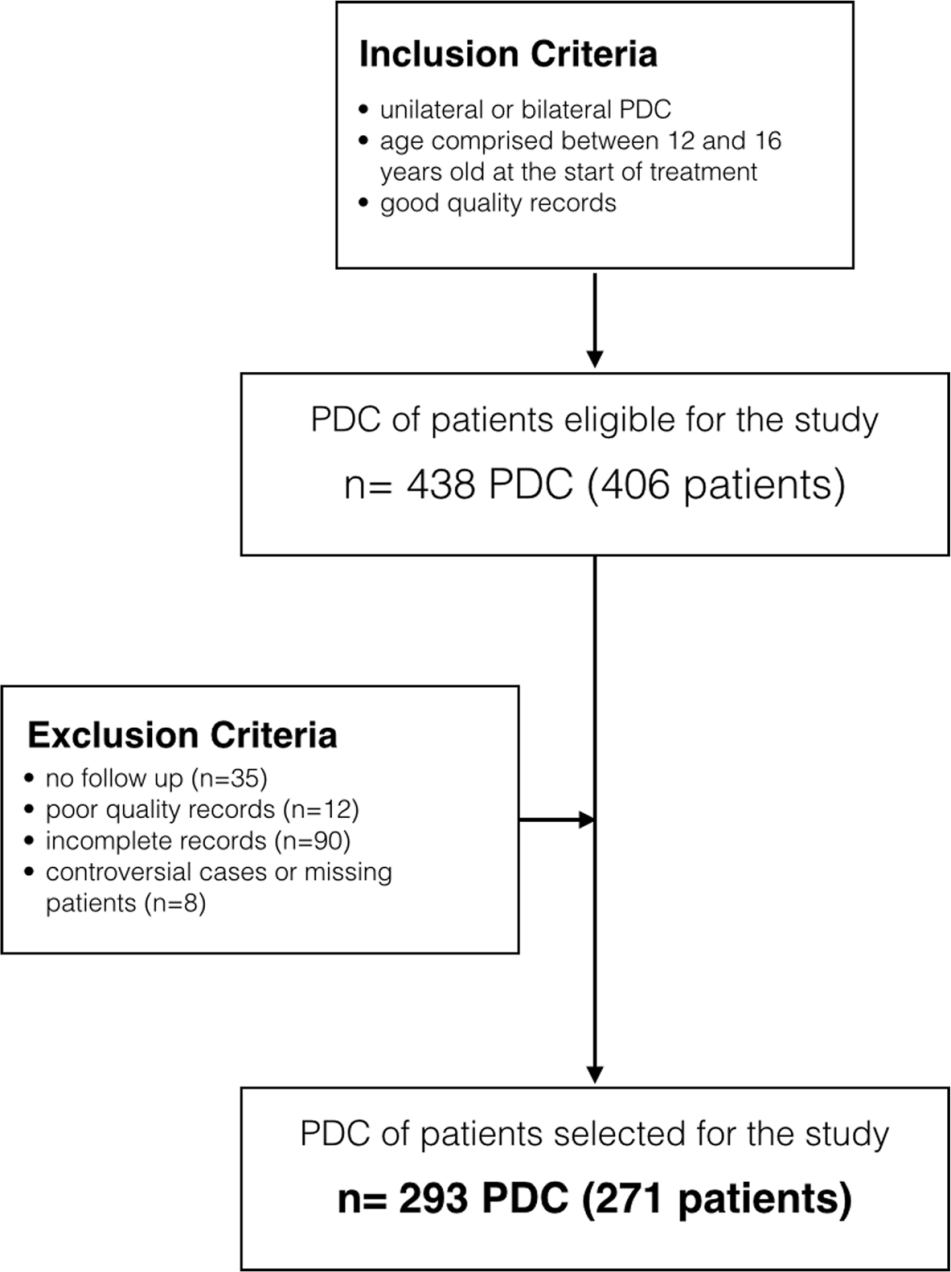
Flowchart for patients’ selection with inclusion and exclusion criteria

The inclusion criteria were as follows: (i) unilateral or bilateral PDC, (ii) age comprised between 12 and 16 years old at the start of treatment, and (iii) good quality records. The exclusion criteria were as follows: (i) no follow-up (35), (ii) poor quality records (12), (iii) incomplete records (90), and (iv) controversial cases or missing patients (8). From the initial sample of 438 impacted PDC, the final sample selected 293 PDC of 271 patients which satisfied the inclusion and exclusion criteria (Fig. 1). The degree of impaction was not considered at this first stage since the aim was to test all different rates of impaction.

An initial evaluation was performed before the surgical treatment of the PDC (mean age 13.8 ± 1.2) and comprised classic orthodontic records (plaster models, pictures, panoramic X-ray, lateral cephalometry). All the patients underwent consecutively the same standardized treatment strategy.

### Surgical procedure and orthodontic device for traction of the impacted canines

All the canines were exposed using CT by the same oral surgeon and orthodontic traction was applied using the “easy cuspid” device [21], in order to move the impacted PDC towards occlusal plane. The easy cuspid appliance was suggested as modification of the Jones Jig appliance, originally developed for molar distalization, and combined with the original idea of Jacoby’s ballista spring [4]. The major difference is a soldered double terminal with a larger end for insertion into the molar band’s headgear tube and a smaller end for the auxiliary tube. A triple-tube molar band is used so that a stabilizing wire can be inserted into the main archwire tube, providing solid anchorage for the traction system [21].

After exposure and traction, canines were aligned by fixed appliance in both arches, according to Roth’s prescription with an overall mean treatment duration of 23.12 ± 6.2 months. Every patient underwent posttreatment examination of the periodontal status health 4 weeks after fixed appliance removal. Image analysis and periodontal status evaluation were performed for all PDCs as follows.

### Image analysis

**Fig. 2:**
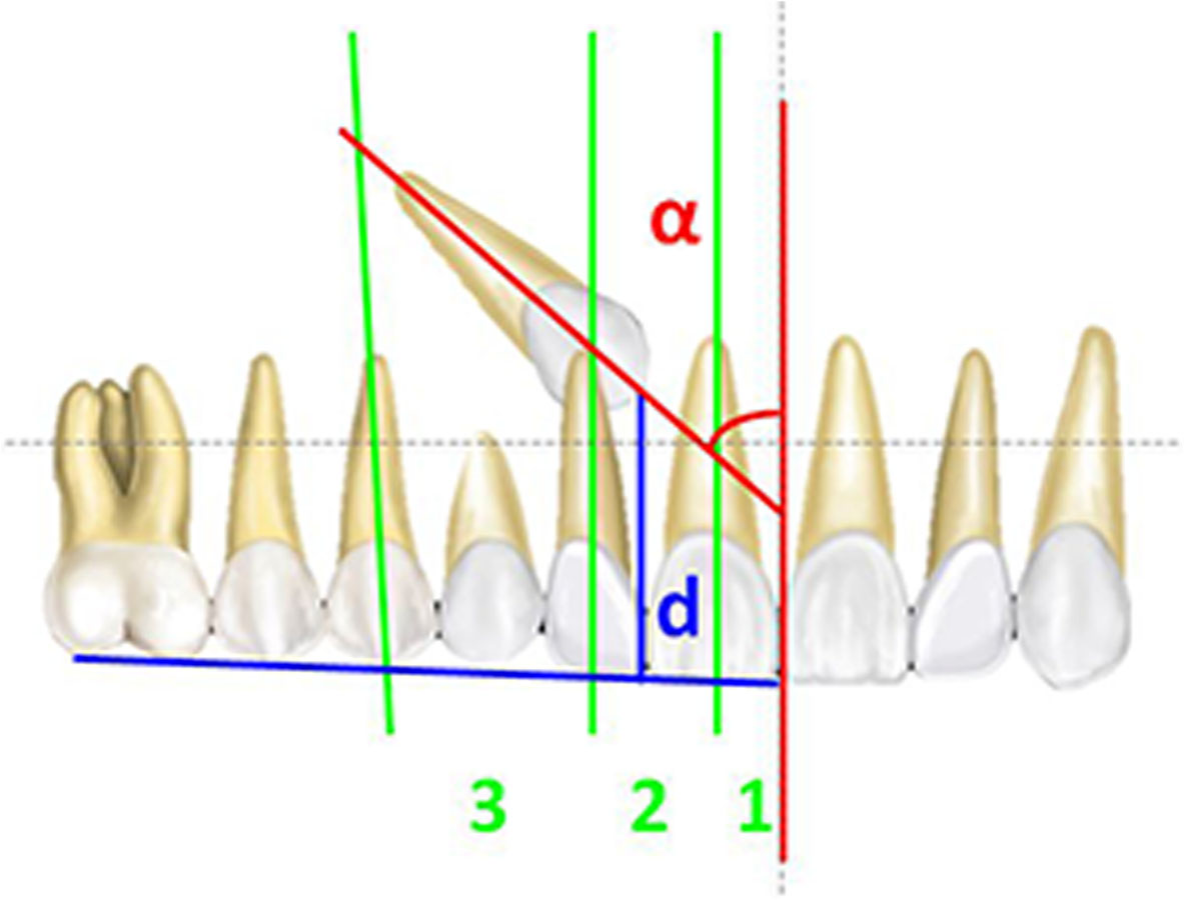
Measurements for canine diagnosis on panoramic X-ray. Blue line, occlusal plane; red line, midline

Panoramic X-rays performed with the same X-ray machine and by trained technicians were available at the start of the treatment (T1) for all patients. The radiographic parameters that were analyzed to assess the position according to a modified version of Ericson and Kurol’s criteria [2, 20, 21] (Fig. 2) of the PDC were as follows:

- *Sector (S):* area where the cusp of the PDC was located (sector 1: between the inter-incisor median line and the long axis of the central incisor; sector 2: between the long axes of the lateral and central incisors; sector 3: between the long axes of the lateral incisor and the first premolar);
- *α-Angle:* the angle formed between the long axis of the impacted canine and the inter-incisor median line (normal value = 20–53°);
- *Distance (d):* the distance between the peak of the impacted cuspid and the occlusal plane (normal value = 7–26 mm).

### Periodontal measurement

The periodontal health was evaluated at the end of the treatment (T2) by the measurements of the following variables:

- *Probing depth* (PD): the depths of the pocket were measured as the distance between the free gingiva margin to the bottom of the pocket with a standard periodontal probe (Williams probe to an accuracy of 0.5 mm). PD was recorded as the mean at six sites (mesiobuccal, midbuccal, distobuccal, mesiopalatal, midpalatal, and distopalatal);
- *Bleeding index* (BI): the buccal surface was scored on a scale of 0 to 1.

After the measurements, all the canines included in the study were divided into three groups according to the PD: PD ≤ 2 mm (group 1); PD > 2 mm (group 2); and CEJ visible (group 3). Such procedure allowed to compare all the other parameters among the three groups.

#### Method error

Tracings on panoramic X-rays were repeated on 25 subjects randomly selected with a month interval by the same trained operator. Intraobserver reproducibility for the image analysis was measured using intraclass correlation coefficient (ICC) for the identification of sector (*S*), *α*-Angle, and distance (*d*).

#### Statistical analysis

Sample size was first calculated before selecting the subjects on the basis of previous evidence [21]. To retrieve beta = 0.80 with alpha set at 0.05, a sample of at least 200 subjects was necessary. Dropout patients should be considered for the retrospective design of the study, then a greater number of patients was enrolled in order to be sure to have at least 200 patients in the final sample. The SPSS software, version 13.0 (SPSS® Inc., Chicago, IL, USA) was employed to perform the statistical analysis. The Shapiro-Wilk test confirmed the normal distribution of the data for the tested sample, then parametric tests were used. Means and standard deviations were computed for all the tested variables, and one-way analysis of variance (ANOVA) was used to assess the significance of the differences among the groups according to PD. When significant interactions were seen, *t* test was employed for pairwise comparisons among all the possible combinations of the groups (Table 2). A *p* value less than 0.05 was used in the rejection of the null hypothesis. A Pearson rho correlation coefficient was then employed to evaluate the strength of the relationship between several tested parameters (*S*, *α*-Angle, and *d*) after treatment, respectively, and the PD. Finally, the multiple backward linear regressions were used to estimate association of each tested parameters (explanatory variables) with the PD.

## Results

Intraclass correlation coefficients (*r*) ranged from 0.95 (*d*-distance) to 0.99 (*S*-sector).

Demographic information of the sample is shown in Table 1. Mean and standard deviations (SDs) were computed for all tested variables as shown in Table 2. According to PD, the sample was divided into three groups, as previously described. The three groups had different sample sizes due to the fact that the prevalence of PD > 2 mm and of CEJ visible was significantly lower than the prevalence of PD < 2 mm. Bleeding index (BI) showed very low values for all the groups and the differences among them were not significant. Similar results were found for the variables *α*-Angle and *d*, since the scores showed similar results for all the groups with no significant differences, even when the variability of the scores was higher such as for *α*-Angle where the range was between 19° and 22.67°. The only tested variable showing significant differences was *S*, since canines with CEJ visible at the end of the treatment presented sectors with a mean score of 1.67, which was significantly different when compared to group 1, where the mean *S* was of 2.36 for the canines that showed PD < 2 mm at the end of the treatment.

**Table 1:** Description of the sample

	Mean	SD	Minimum-maximum
Age at the beginning of treatment (years)	13.8	1.2	12.6–15
Treatment time (months)	23.12	6.2	16.9–29.32

Means and SDs for age at the beginning of treatment and treatment time

**Table 2:** Variables are shown as means and SDs

Group (*n*)	Probing depth (PD)	Bleeding index (BI) (score 0 = no; 1 = yes)		Sector (S1, S2, S3)		*α*-Angle (°)		Height (H2, H3, H4)	
		Mean	SD	Mean	SD	Mean	SD	Mean	SD
1 (274)	PD < 2 mm (1;2)	0.51	0.50	2.36	0.75	19.00	10.87	3.09	0.81
2 (13)	PD > 2 mm (3;4)	0.43	0.62	1.79	0.84	16.50	11.31	2.71	0.84
3(6)	CEJ visible	0.33	0.82	1.67^*^	0.75	22.67	11.31	2.83	0.75

^*^Significant when compared to group 1

Even though *S* was the only tested variable showing significant differences among the groups, when tested at the linear regression (Table 3), none of the tested parameters (*S*, *α*-Angle, and *d*) after treatment showed significant correlations with PD suggesting a very low influence of these parameters (*R* square of the model = 0.12) on the variation of PD at the end of treatment.

**Table 3:** Results of the multiple backward linear regression to estimate association of the tested parameters with the PD after treatment (*n* = 293)

Explanatory variable	*β*	*t*	*P*
Sector	−0.016	−0.281	0.779
*α*-Angle	0.006	0.100	0.920
Height	0.108	1.841	0.067

β correlation coefficients; R square of the model, 0.12. None of the differences in the tested parameters was significantly associated with PD scores after treatment at the multiple linear regression

## Discussion

The purpose of the present retrospective study was to assess the influence of initial position on posttreatment periodontal health of PDC treated by a combined surgical-orthodontic approach using closed flap technique. The considered radiographic variables (*S*-sector, *α*-Angle, *d*-distance) were suggested by Ericson and Kurol [2] with a different diagnostic and prognostic significance according to the phase of development of the dentition estimating the possibility of successful early treatment and combined orthodontic-surgical treatment in patients with PDC [22–24]. According to the present results, the only tested variable showing significant differences in periodontal health outcome of examined PDC was the *S*-sector. Crescini et al. [20] previously investigated the pretreatment radiographic features for the periodontal prognosis of treated impacted canines and found no significant influence of *S*-sector, *α*-Angle, and *d*-distance on the periodontal outcomes. Nevertheless, the tested variables resulted significant and useful in the prediction of the duration of active orthodontic traction with precise indications on the variability of the prediction parameters. Stewart et al. [25] also found that overall treatment duration was affected by the *d*-distance of the impacted canine from the occlusal plane, and Zuccati et al. [24] reported that the amount of chairside time in patients with impacted canines was proportional to the patient’s age, the *d*-distance, and the *S*-sector, and it was inversely proportional to the *α*-Angle of impaction. The present study replicated similar methods if compared to the study of Crescini et al. [20], but taking into consideration higher sample of patients and using a different traction device. The results, in fact, were only partially similar to the previous study. Indeed, similar results were detected when normal (PD < 2 mm) or slightly increased PD (PD > 2 mm) was investigated. In those groups of patients, in fact, no significant differences were detected in terms of *S*-sector, *α*-Angle, or *d*-distance. In the study of Crescini et al. [20], only one patient presented with a gingival recession of 1 mm at the final observation, whereas in the present study, six patients exhibited CEJ visible due to recession. This difference might be related to the bigger sample of the present study and allowed to retrieve significant difference in relation to the *S*-sector in patients with CEJ visible when compared to normal or slightly increased PD. According to the present results, *α*-Angle and *d*-distance seem to not influence the periodontal outcome of the treated impacted canine regardless of the amount of gravity. On the contrary, *S*-sector might cover a significant role when higher rates of gravity are present suggesting the possibility in few cases for periodontal damage at the end of treatment. Considerations on treatment duration were not in the aims of the present study and this lack might be considered as a limit.

In the present study, another variable of periodontal outcome was investigated along with PD and it was BI-bleeding index, which resulted in very low and not significant rates after treatment. This index is not only related to the periodontal attachment but might also give information on the oral hygiene status of the patients that was under strict observation for all the patients selected for the present sample.

The closed flap technique was performed for all the canines treated in the present study by the same surgeon. In literature, the controversy is still open regarding the periodontal outcome of open or closed surgical exposure and subsequent orthodontic alignment of the PDC [10, 11]. Reported periodontal problems included loss of alveolar bone height, increased pocket probing depths, and loss of attached gingivae [18]. Several authors tended to believe that open flap technique might be detrimental in terms of periodontal outcome if compared to closed technique. On the contrary, some authors suggested lower odds of ankylosis of open flap than closed flap and indicated this possible effect as possible direct effect due to trauma of the periodontal ligament or root cementum by the low-speed bur, chemical trauma from the etching gel [10], or the use orthodontic forces of high magnitude or inappropriate direction. Reports form recent trial suggested no significant differences between the two surgical techniques in terms of periodontal outcomes [18]. As regard for the orthodontic traction employed in the present study, the easy cuspid device was used for all the patients, allowing for a predicable amount of force system during traction with both rigid and extended anchorage system [21].

Limits of the present study might be related to the lack of comparison of periodontal health status of treated PDC with spontaneously erupted contralateral canines [26, 27]. Nevertheless, Quirynen et al. [28] and Crescini et al. [20] suggested no significant differences in the periodontal parameters considered between contralateral and impacted canines according to their results. Moreover, only two periodontal variables (probing depth and bleeding index) were employed in the present study as measurements of periodontal health status of treated PDC. Some other variables might definitely have been useful in the correct evaluation of the periodontal health status such as plaque index, attached gingiva, crown length, gingival abnormality, and alveolar bone levels. This might be considered as a limit of the present study; nevertheless, the use of such a big data pool might have been confusing in the interpretation of results since greater number of variables should have been analyzed and discussed and for this reason, only two variables were considered.

The present study was carried out with image analysis performed on panoramic X-rays, even though cone beam computed tomography (CBCT) nowadays represents an exciting new tool for canine impaction diagnosis and treatment planning [29]. According to a recent systematic review [30], CBCT was suggested as more accurate than conventional radiographs in localizing maxillary impacted canine, even though no robust evidence was found to support using CBCT as a first-line imaging method for impacted maxillary canine evaluation, but it is indicated when conventional radiography does not provide sufficient information.

## Conclusions

According to the present results, the following conclusions could be drawn when considering palatally displaced maxillary canine treated with closed technique:

- Radiographic variables as *α*-Angle and *d*-distance seem to not influence the periodontal outcome of treated impacted canine regardless of the amount of gravity;
- *S*-sector might play a significant role when higher rates of gravity are present suggesting the possibility in few cases for periodontal damage at the end of treatment.

The present results should be limited to palatally displaced maxillary canines, since buccally displacement might involve different variables and outcomes as well as different treatment choices and success rate.
